# The inhibitory effect of apatinib on different small cell lung cancer cells and in lung cancer‐bearing mice and patients

**DOI:** 10.1111/crj.13738

**Published:** 2024-02-25

**Authors:** Mingtao Liu, Hui Li, Ranran Guo, Xia Yu, Yu Li

**Affiliations:** ^1^ Department of Pulmonary Medicine Binzhou People's Hospital Binzhou Shandong China; ^2^ Department of Pulmonary Medicine Penglai Traditional Chinese Medicine Hospital Yantai Shandong China; ^3^ Department of Pulmonary Medicine, Qilu Hospital Shandong University Jinan Shandong China

**Keywords:** apatinib, lung cancer‐bearing mice, patients, small cell lung cancer

## Abstract

**Purpose:**

To observe the inhibitory effect of the small molecule tyrosine kinase inhibitor apatinib on small cell lung cancer in vitro and vivo.

**Material and methods:**

Three small lung cancer cells were selected CCK‐8 and monoclonal assay was used to determine the effect of apatinib on proliferation. The effects on cell cycle and apoptosis were detected by flow cytometry and TUNEL. We observed the inhibitory effect of different doses of apatinib on xenograft tumor. The efficacy and safety of apatinib in 20 patients with advanced small cell lung cancer were observed.

**Results:**

For small cell lung cancer with high expression of VEGFR2, apatinib has a significant inhibitory effect both in vitro and in vivo. It can play an inhibitory role by promoting apoptosis and cell cycle arrest pathways. For small cell lung cancer with low expression of VEGFR, the inhibitory effect on cells in vitro was not significant. It has certain inhibitory effect on nude mouse transplanted tumor and small cell lung cancer patients, but the effect is weaker than that of animal models and patients with small cell lung cancer cells with high expression of VEGFR2.

**Conclusion:**

Apatinib has a significant inhibitory effect on small cell lung cancer with high expression of VEGFR2 and may be a treatment for small cell lung cancer patients.

## Graphical abstract

Apatinib inhibited the proliferation of VEGFR‐2 high‐expression small lung cancer cell lines H446 and H69 in a concentration‐dependent manner. In vivo studies showed that both H446 and H69 xenografts were significantly inhibited by low‐ and high‐dose apatinib. Apatinib also has some efficacy in patients with small cell lung cancer.

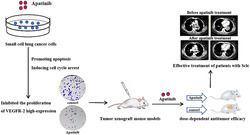

## 1 INTRODUCTION

The incidence and mortality rate of lung cancer were high,^1^ and the fatality rate were high, especially small cell lung cancer (SCLC).^2^ At present, there is still no effective treatment for SCLC.^3^ No specific target genes have been identified, so there are no effective targeted drugs for SCLC.^4^ Etoposide combined with cisplatin is still the first‐line treatment for SCLC.^5^ Although combined radiotherapy can improve the survival of patients with SCLC to some extent, the short‐term recurrence rate is very high, and the overall efficiency of irinotecan as a second‐line treatment was low.^6^ There is necessary to find more effective treatments against SCLC.^7^

The growth of tumor needs new blood vessels, blocking the blood vessels of tumor, can achieve the purpose of tumor control.^8^ According to different mechanisms of action and targets, the current commonly used antiangiogenic drugs are mainly classified into three categories.^9^ Monoclonal antibodies to VEGF, of which the representative drug is bevacizumab, which can bind VEGF‐A, inhibit its binding to VEGF receptor‐2 and subsequently inhibit the biological effects of VEGF. The other is rh‐endostatin, which suppresses tumor neovascularization by inhibiting endothelial cell migration. Another type is the small molecule multi‐target tyrosine kinase inhibitors, represented by drugs such as anlotinib and apatinib, which mainly acts on VEGF‐VEGFR signaling pathway and inhibition of the tumor neovascularization. At present, antiangiogenic drugs mainly act on VEGF–VEGFR, because they play a decisive role in the process of tumor neoangiogenesis.^10^ In particular, the VEGFA–VEGFR2 signaling pathway is the most important pathway.^11^ Apatinib suppresses the generation of tyrosine kinase by highly selectively competing for ATP binding sites in VEGFR‐2 and thus inhibiting the generation of new blood vessels in tumor tissues.^12^ Previously, apatinib was approved in China in 2014 for the treatment of advanced gastric cancer.^13^ Clinical studies on apatinib in the treatment of advanced lung cancer have also confirmed that it has a certain effect, mainly for advanced non‐SCLC, and there are few clinical studies on SCLC. There is a lack of randomized controlled studies.^14^ In our previous study, we found that VEGFR2 showed a certain degree of expression in SCLC tissues, but there were few reports on the basic and clinical of apatinib in the treatment of SCLC. We selected three types of lung cancer cells, through cell test, constructed animal transplant tumor model, and a minority of SCLC patients, to observe the inhibitory effect and pathway of apatinib on SCLC.

## 2 MATERIALS AND METHODS

### 2.1 VEGFR2 expression with prognosis

Tissue specimens of 40 patients with SCLC were randomly selected and all specimens were obtained by bronchoscopy and percutaneous lung puncture biopsy. The expression of VEGFR2 in the samples was detected by immunohistochemistry and divided into high expression group and low expression group according to the staining intensity and positive proportion. The patients in the two groups were followed up to obtain the PFS and OS of the patients, and the relationship between the prognosis of patients with SCLC and VEGFR2 expression was observed.

### 2.2 Cell culture and compounds

Human lung cancer cell lines H446, H69 and H524 were purchased from the Cell Bank of the Chinese Academy of Sciences (Shanghai, China). The previous studies have confirmed that the expression levels of VEGFR2 in the three types of SCLC cells were differed significantly, both in high and low expression. It is possible to observe the relationship between the expression level of VEGFR2 and the ability of apatinib to inhibit proliferation in a representative manner. All cell lines were cultured in RPMI‐1640 medium supplemented with 10% fetal bovine serum (Gibco, USA) at 37°C in 5% CO_2_. Apatinib was obtained from Selleck (s2221, Shanghai, China), dissolved in DMSO and diluted with 1640 medium for in vitro studies. Apatinib mesylate was obtained from Hengrui Medicine Co. Ltd. (Jiangsu, China) for animal testing. The primary antibodies include c‐caspase3, ki67, CD31, and β‐actin were purchased from abcam, while the antibodies of VEGFR2, P53, and cyclinB1 were get from CST. The secondary antibodies were supplied from Wuhan Sanying.

### 2.3 Cell viability assay and colony formation assay

About 2000 cells were added to each well with about 100 μl, four to six multiple wells were set up in each group, and a blank group was set up at the same time. PBS buffer was added to the most marginal hole, and the cells were cultured in a 5%CO_2_ cell incubator at 37°C for 12 h. The medium with different concentrations of drugs was added to the experimental group, and the medium without drugs was added to the blank group and then incubated in a cell incubator with 5% CO_2_ air at 37°C for 24–72 h. CCK‐8 solution was added and incubated in a 5%CO_2_ incubator at 37°C for 2 h. OD values of each hole at 450 nm wavelength were detected by enzyme‐labeled instrument, data were analyzed and processed, proliferation curves were drawn, and IC_50_ values were calculated.

About 500 cells/wells were inoculated in the six‐well plate culture plate, and media containing different concentrations of apatinib were added and cultured in a 5%CO_2_ cell incubator at 37°C for 14 days. The medium was changed every 3 days during the process and cell status was observed. After the completion, it was washed with PBS solution, fixed with 4% paraformaldehyde, dyed with crystal violet, and photographed after PBS washing. Each group of data was presented as mean ± SD.

### 2.4 Apoptosis analysis and cell cycle by flow cytometry assay

After the cells were treated with apatinib at different concentrations, the culture medium was removed, and pancreatic enzyme was added for digestion in the incubator. Three milliliters of culture medium containing serum was added to terminate pancreatic enzyme digestion. The cells were centrifuged at 1000 rpm for 5 min at room temperature, the cells were suspended by PBS, the cells were centrifuged at 1000 rpm for 5 min at room temperature, and the fixed cells were suspended by 75% alcohol and placed in the refrigerator at 4°C overnight. Centrifuge at 1000 rpm for 5 min at room temperature, remove the supernatant, add PBS for three times, add PI staining solution, stain at 37°C for 30 min, and then detect by flow cytometry.

Procedure for apoptosis detection: After cell intervention, Annexin V and PI staining solution were added, gently mixed, incubated at room temperature without light for 15 min, and centrifuged at 1000 rpm for 5 min at room temperature, the supernatant was removed, and the cells were suspended by PBS and immediately detected by flow cytometry. Data analysis depends on the FlowJo X software.

### 2.5 Assessment of apoptosis by TUNEL and cell immunofluorescence

The TUNEL cell apoptosis detection kit uses a one‐step staining method to rapidly detect cell apoptosis. After the intervention of apatinib with different concentrations, the cells were washed and fixed and incubated at room temperature for 5 min with permeable solution, then washed again with 50 μl TUNEL working solution, incubated at 37°C for 60 min without light, and washed with PBS for three times. The anti‐fluorescence quenching sealing solution was added, and the fluorescence effect was observed under fluorescence microscope.

For immunofluorescence for the apoptotic protein caspase‐3, the specific method is as follows: the slides of crawled cells were washed with PBS for three times in the culture plate, 4% paraformaldehyde fixed in climbing sections, permeabilized with 0.5%Triton X‐100 for 20 min at room temperature. Add goat serum on glass slides, closed for 30 min at room temperature. With the addition of the c‐caspase‐3 antibody, 4°C overnight, fluorescent secondary antibodies were added the following day and incubated for 1 h at 37°C in the wet box, DAPI was incubated for 5 min, and the specimens were stained with nuclei. Seal fluid containing anti‐fluorescence quencher and then the collected images were observed under a fluorescence microscope.

### 2.6 Western blot analysis

Protein was extracted from cells and tissues and denatured in boiling water bath, DNA was broken by ultrasound and centrifuge, and the supernatant containing total protein was obtained. The protein concentration was detected by BCA method, and the sample amount was calculated. After the protein was isolated by polyacrylamide gel electrophoresis (SDS‐PAGE), the protein was transferred to PVDF membrane and sealed by sealing solution, the primary antibody and the secondary antibody were added respectively, and the color was developed by DAB. The protein bands were detected using Invitrogen Bright system.

### 2.7 RNA preparation and RT‐PCR analysis

The primers for VEGFR2 were designed with reference to the literature, and GAPDH was used as the internal reference. Total RNA was extracted by Trizol according to the instructions of the kit. The purity of RNA was measured by microspectrophotometer. The RNA was reversely transcribed into cDNA according to the instructions of reverse transcription kit. SYBR dye method was used for amplification and detection, and Ct values were derived for analysis.

### 2.8 Tumor xenograft mouse models

About 200 μl of 3 × 10^6^ lung cancer cell suspension was injected into the right chest wall of nude mice aged 4–6 weeks. When the transplanted tumor volume reached 50 mm^3^, drug intervention was performed. They were randomly divided into three groups, which were given normal saline, apatinib 80 mg/kg and 120 mg/kg gavage, respectively. The long and short diameter of the transplanted tumor was measured every 3 days, the tumor volume was counted by (length × width^2^/2), and the tumor growth curves were plotted. The nude mice were sacrificed on the 21st day, and transplanted tumor tissue was obtained.

### 2.9 Immunohistochemical staining

The tissues were embedded with paraffin wax, and the sections with thickness of 4 μm were prepared, dewaxed, and hydrated at room temperature. Heat antigen repair; 3% hydrogen peroxide inactivates endogenous enzyme activity. Add goat serum sealer and put in a wet box at room temperature for 10 min. Shake off the sealing liquid, add the primary antibody, and then put in the wet box at 4°C overnight. On the second day, add the second antibody and DAB for color development. The positive cells were those with obvious yellow or brown‐yellow particles in the cytoplasm or nucleus. Each section was subjected to a microscope image analyzer, and five complete nonoverlapping visual fields in the positive cell area were randomly observed under a 400‐fold light microscope, and the staining intensity was observed.

### 2.10 Patients, treatment, and evaluation

Twenty patients with SCLC with a definite pathological diagnosis from April 2018 to August 2020 in our hospital were recruited. After first‐ or second‐line chemotherapy and local radiotherapy, the lesion progressed with poor results. The patients were treated with third line and above, in poor health, with PS score 1–3. The histopathological immunohistochemistry showed that the VEGFR2 was highly expressed in more than 50% of 17 patients, and other three patients were low expression. The basic conditions of the patients and the expression of VEGFR2 are shown in Table 1. In application of apatinib as a third‐ or above‐line therapy regime that apatinib (750 mg/day) was administered until severe adverse reactions occur, the continuation of oral medication cannot be tolerated. All patients underwent a spiral CT of the chest to calculate the size of the lesion before and 1 month after the administration of apatinib. At least one measurable lesion according to Response Evaluation Criteria in Solid Tumors, version 1.1 (RECIST v1.1).

**TABLE 1:** Patient characteristics and responses.

No.	Gender	Age years	Stage	PS score	Previous treatment regimen	Toxicity 3/4 grade	Efficacy	PFS (m)	OS (m)	Expression VEGFR2 (%)
1	Male	59	Limited	1	EP; RT	No	SD	4.4	6.5	65
2	Male	65	Extensive	2	EP; IC;	No	SD	2.5	5.6	57
3	Female	52	Extensive	2	EP; IC; TP	No	SD	2.9	5.1	52
4	Female	72	Limited	2	EP; RT; TP	Fatigue	SD	3.3	6.1	58
5	Male	73	Extensive	3	EP + RT; IC	No	PD	2.0	3.5	32
6	Male	63	Limited	2	EP + RT; IC; TP	No	PR	4.9	6.5	69
7	Female	47	Extensive	1	EP; TP	No	SD	3.8	5.4	51
8	Female	55	Extensive	2	EP; IP; TP	No	SD	4.5	5.8	55
9	Female	53	Limited	1	EP + RT	Hypertension	SD	2.9	4.3	57
10	Female	63	Limited	2	EP; IC; RT	No	SD	3.2	6.5	59
11	Male	69	Limited	2	EP + RT	Proteinuria	SD	3.4	5.8	61
12	Male	72	Extensive	2	EP; IC	No	SD	4.1	5.2	51
13	Male	53	Extensive	1	EP; IP	No	SD	3.9	6.3	55
14	Female	49	Extensive	3	EP; TP	No	PD	2.1	3.5	21
15	Male	66	Limited	2	EP + RT; IC	No	SD	3.3	5.5	52
16	Male	72	Extensive	2	EP; IC	Fatigue	SD	4.1	6.2	55
17	Male	70	Limited	2	EP + RT; TP	No	SD	3.9	5.8	58
18	Female	55	Limited	2	EP; IC; RT	Hypertension	SD	3.2	4.4	50
19	Female	58	Extensive	3	EP; IP	No	PD	1.7	2.9	18
20	Male	61	Extensive	1	EP; TP	No	SD	2.8	4.6	57

Abbreviations: EP, etoposide + cisplatin; IC, irinotecan + carboplatin; IP, irinotecan + cisplatin; OS, overall survival; PD, progressive disease; PFS, progression‐free survival; PR, partial response; RT, radiation therapy; SD, stable disease; TP, topotecan + cisplatin.

### 2.11 Statistical analysis

The data were statistically processed by SPSS21.0 software. *T*‐test was applied for data comparison between the two groups. Two‐way ANOVA was used for data pairs between the three groups. Statistical differences were considered as *P* < 0.05.

**FIGURE 1:**
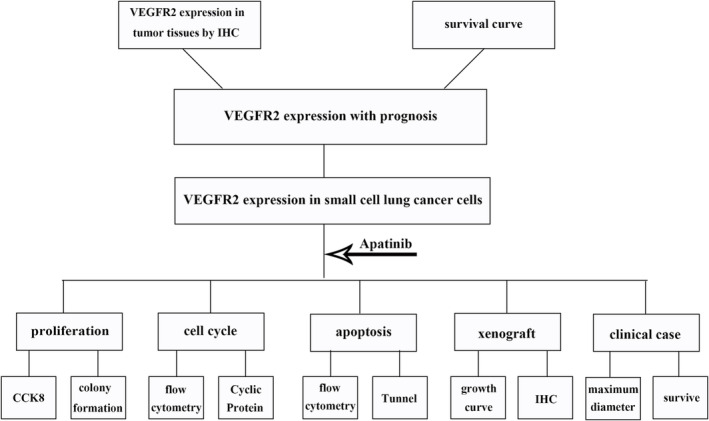
The flowchart of the study.

The flowchart of this study is shown in Figure 1.

## 3 RESULTS

### 3.1 VEGFR2 expression with prognosis

**FIGURE 2:**
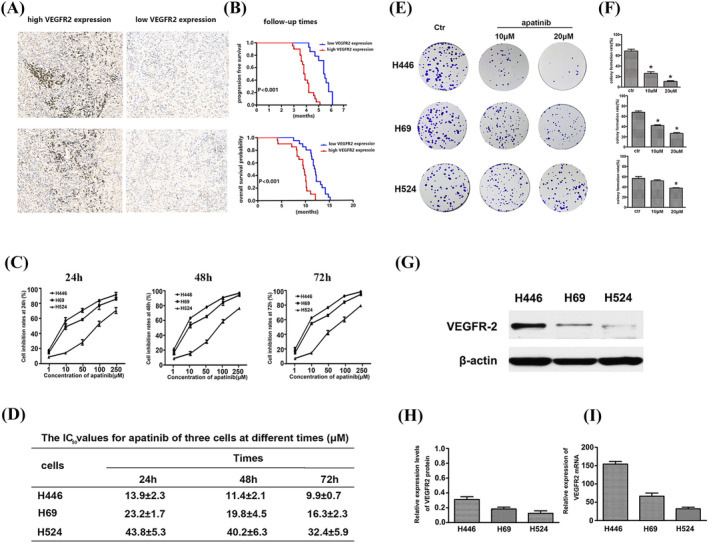
(A) The VEGFR2 expression was detected in the nucleus and cytoplasm. (B) The correlation between the follow‐up times and VEGFR2 level. (C) Inhibition rate of apatinib on three small lung cancer cell lines at 24, 48, and 72 h. (D) IC_50_ values for apatinib of three cells at different times. (E,F) Effect of apatinib on colony formation of three cells. (G,H) Relative expression levels of VEGFR‐2 protein. (I) Relative mRNA levels of VEGFR‐2 in three cell lines.

The expression of VEGFR2 in SCLC tissues is shown in Figure 2A. Tissues with high VEGFR2 expression on the left and low VEGFR2 expression on the right. The PFS and OS of the two groups were shown in Figure 2B. The results showed that SCLC patients with high VEGFR2 expression had worse prognosis, and the median PFS and OS were significantly lower than those with low VEGFR2 expression, with a statistically significant difference (*P* < 0.001).

### 3.2 Antiproliferative activity against small lung cancer cells

To detect the inhibition rate of apatinib in three SCLC by CCK8 method. Result display that with the increasing concentration of apatinib, the inhibition rate of the three lung cancer cells gradually increased. Among them, the inhibition of H446 and H69 cells was more significant, and it was stronger in inhibiting cell proliferation than that in H524 cells. The inhibition rate of apatinib was 50 μM at 24 h, and the inhibition rate of H446 and H69 was 67.4 ± 4.32% and 53.35 ± 3.18%, while the inhibition rate of H524 was only 22.45 ± 5.12%, showing significant statistical difference (*P* < 0.05) (Figure 2C). Over time, the IC_50_ of apatinib in all three lung cancer cells decreased. Comparing the three lung cancer cells, the IC_50_ value of apatinib for H446 and H69 was significantly lower than that of H524 cells (*P* < 0.05) (Figure 2D). With the increasing concentration of apatinib, the number of cell colonies gradually decreased, and the inhibitory effect on H446 and H69 was more obvious. While for H524 cells, it showed significant inhibition of colony formation only at high concentrations of apatinib (Figure 2E,F).

### 3.3 VEGFR2 protein and mRNA expression in cells

The expression of VEGF R‐2 in three lung cancer cells by western blot showed that H446 cells had the highest expression of VEGFR‐2, followed by H69 cells, and H524 cells had the lowest expression, and there were significant statistical difference (*P* < 0.05) (Figure 2G,H). The level of mRNA measured by qPCR was consistent with the protein expression level. H446 cells showed the highest expression, and H524 cells showed the lowest expression level (Figure 2I).

### 3.4 Cell cycle regulation

**FIGURE 3:**
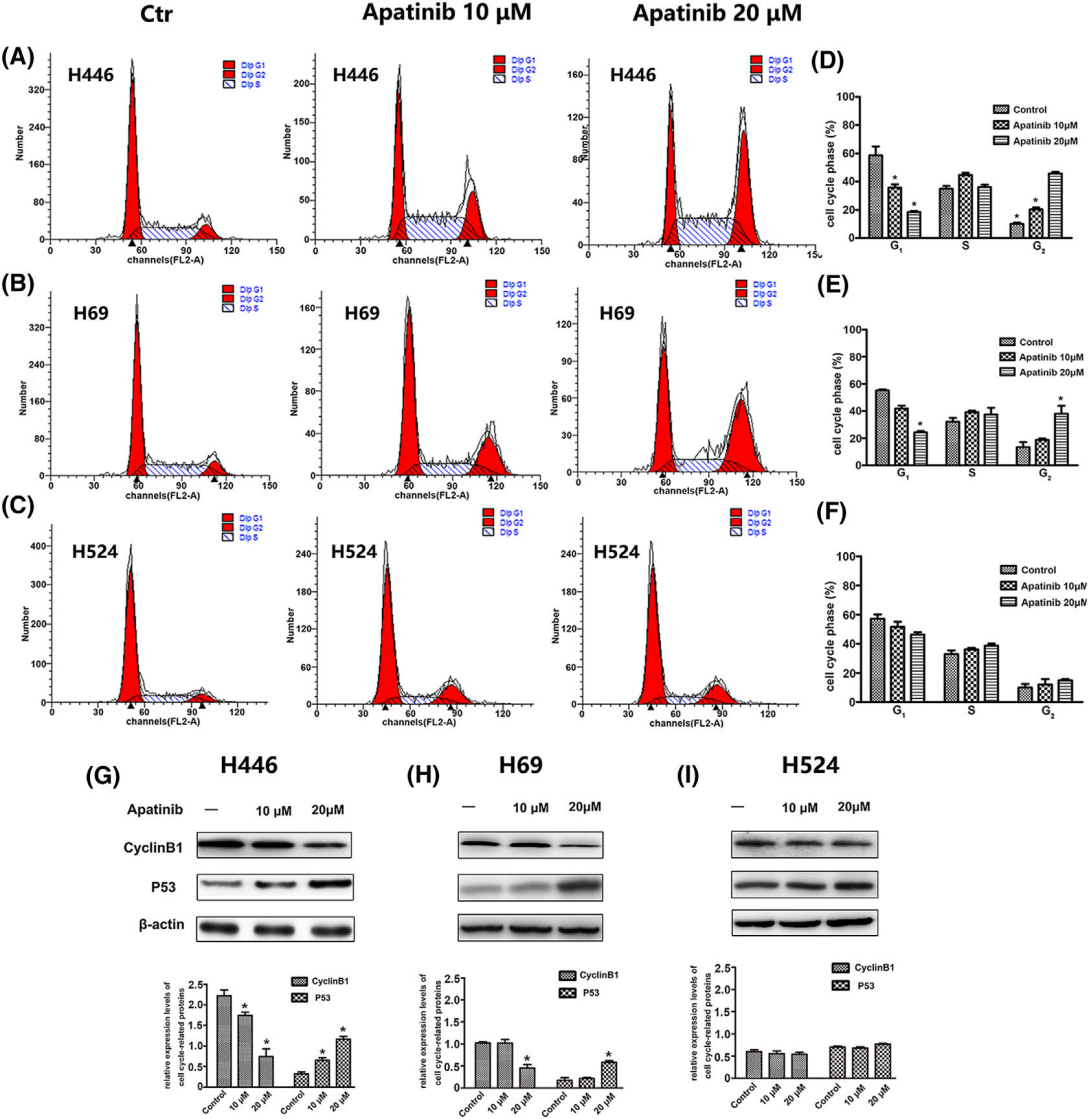
Apatinib induced cell cycle arrest in three cell lines. (A–F) H446, H69, and H524 cells were treated with 0, 10, or 20 μM apatinib for 24 h, and the cell cycle was analyzed by flow cytometry. (G–I) Effect of apatinib on the expression of cyclin B1 and p53 was tested by western blotting in H446, H69, and H524 cells, respectively. Cells were treated with 0, 10, or 20 μM apatinib for 24 h.

Among the three types of cells, apatinib had the most significant cycle arrest effect on H446 cells. Apatinib resulted in a significantly higher proportion of the G2 phase, and the ratio of G2 phase was 11.12 ± 0.80% in control group, while after the addition of 10 and 20 μM apatinib, the ratio of G2 phase increased to 24.33 ± 1.34% and 46.53 ± 1.62% with significant statistical difference (*P* < 0.05) (Figure 3A,D). The similar results were seen in H69 cells (Figure 3B,E). However, for H524 cells with low expression of VEGFR‐2, the arrest effect on G2 phase was not obvious, and the proportion of G2 phase was not significantly increased after the addition of apatinib (Figure 3C,F). To further verify the cycle arrest effect of apatinib on the three cells, the expression of G2 phase specific protein cyclin B1 and P53 was measured by western blot. In H446 and H524 cells, cyclin B1 expression gradually decreased, and P53 expression gradually increased with increasing apatinib concentration (Figure 3G,H). However, the above effect was not found in H524 cells (Figure 3I).

### 3.5 Effect of apatinib on promoting apoptosis

**FIGURE 4:**
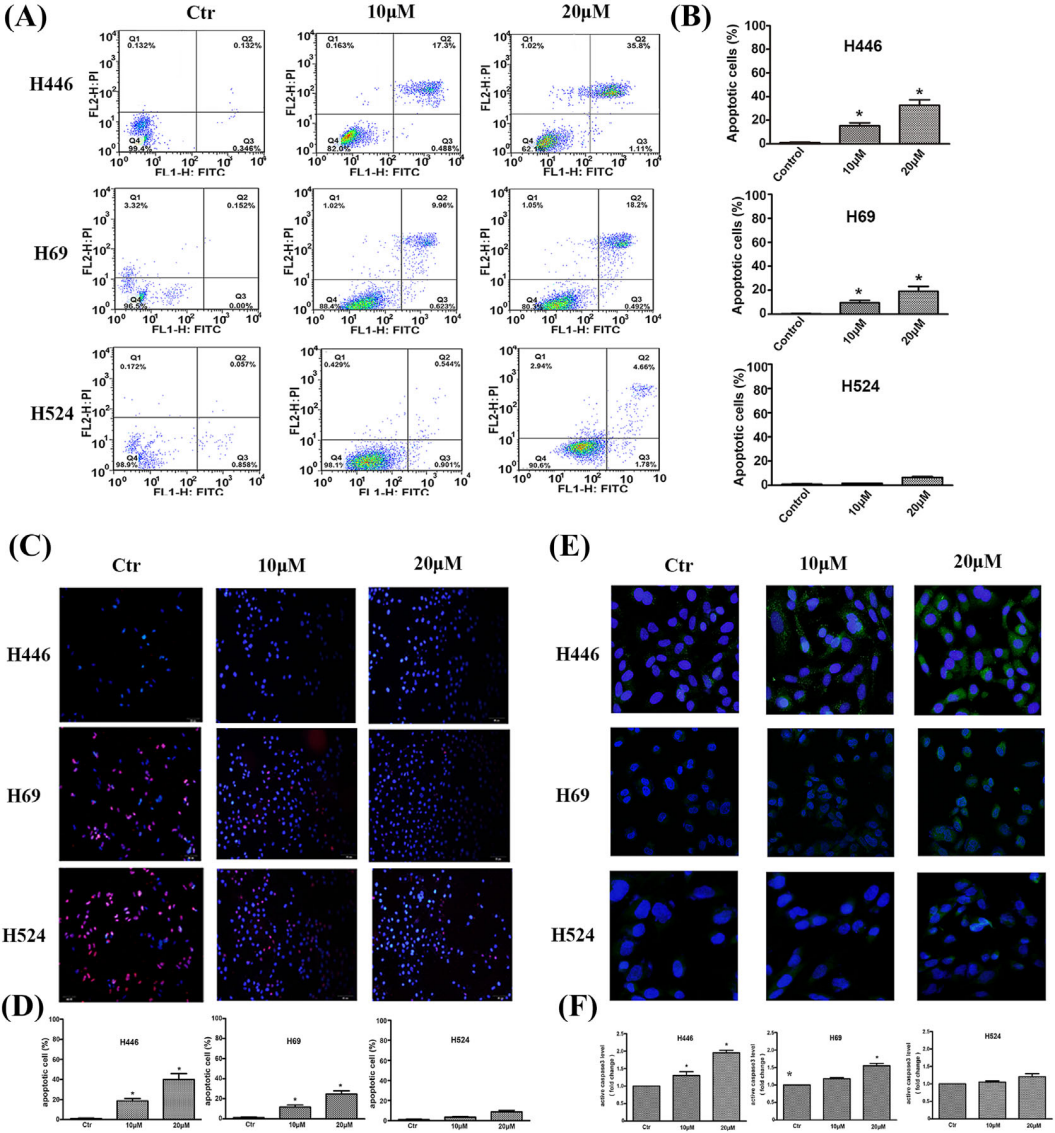
Effects of apatinib on the apoptosis rates of H446, H69, and H524 cells. (A,B) Apoptosis rates for H446, H69, and H524 cells treated with 0, 10, or 20 μM apatinib for 24 h were detected by flow cytometry. (C,D) Effects of apatinib on the apoptosis rates of H446, H69, and H524 cells were detected by TUNEL assay. (E,F) Effects of apatinib on the expression of apoptosis‐related proteins were tested by immunofluorescence.

The effect of apatinib promoted apoptosis in H446 cells were most significant and with a concentration dependence by flow cytometry. The ratio of apoptosis in the 10 and 20 μM groups were 14.7 ± 3.45% and 33.7 ± 4.57%. The results were similar in H69 cells, with apoptosis ratios of 9.8 ± 2.61% and 18.9 ± 2.35%, respectively. However, for H524 cells, its apoptosis was not obvious, and the proportion of apoptosis observed in the 20 μM concentration group was only 6.9 ± 1.85% (Figure 4A,B). The results of tunnel detection of apatinib on apoptosis of three kinds of cells were consistent with those of flow cytometry. The apoptosis effect of H446 and H69 was more obvious, and the apoptosis effect was more significant with increasing concentration. Taking H446 cells as an example, the ratio of apoptosis at concentrations of 10 and 20 μM was 19.7 ± 3.21% and 39.4 ± 3.21%. The effect of apoptosis in H69 cells was slightly weaker than that of H446 cells. However, the effect of promoting apoptosis in H524 cells was not obvious, and the proportion of apoptosis in the 20 μM concentration group was only 8.2 ± 2.81%, which was significantly weaker than that in H446 and H69 cells (Figure 4C,D). By immunofluorescence, apatinib promotes apoptosis through caspase‐3 progression. In H446 and H69 cells, treatment with a certain concentration of apatinib significantly increased active caspase‐3 levels in a concentration‐dependent manner. However, after apatinib intervention, the expression level of active caspase‐3 in H524 cells was not significantly higher than that in the control group (*P* > 0.05) (Figure 4E,F).

### 3.6 Tumor growth and immunohistochemistry

**FIGURE 5:**
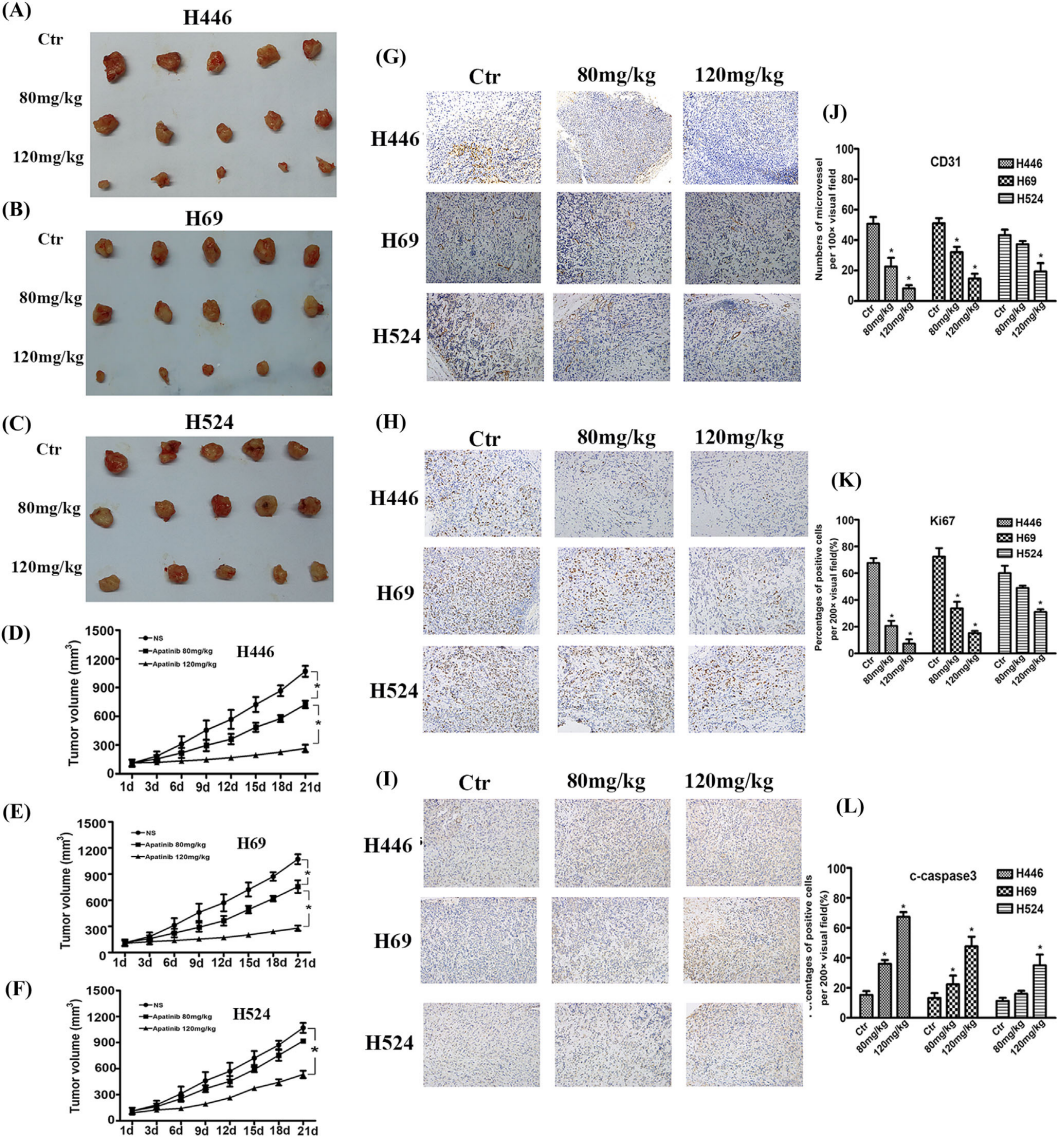
(A–C) BALB/c nude mice xenografted with H446, H69, and H524 cells were treated with vehicle or apatinib (80, 120 mg/kg, po, qd) for 21 days, and the tumor volume of three groups at 21 days. (D–F) Tumor growth curves for three groups. (G–I) Expression of CD31, Ki67, and c‐caspase‐3 in the different groups of xenografted tumor tissues. (J–L) Histogram graph of expression of CD31, Ki67, and c‐caspase‐3 in the different groups of xenografted tumor tissues.

H446 and H69 cell grafted nude mouse tumors, whether 80 or 120 mg/kg apatinib, showed significant inhibitory effect on tumor growth. After 3 weeks of apatinib treatment, the tumor volume was significantly reduced compared with the control group (*P* < 0.05). After 3 weeks of treatment with 80 mg/kg apatinib, there was no significant difference in tumor volume between the nude mice transplanted with H524 cells and the control group (*P* > 0.05). Only the 120 mg/kg group showed a significant inhibitory effect on tumor growth, which was significantly different from the control group (*P* < 0.05) (Figure 5A‐F).

In the transplanted tumor tissues of nude mice constructed with H446 and H69 cells, the expressions of CD31 and Ki67 in tumor tissues were significantly decreased, and the expression of c‐caspase‐3 was significantly increased after the intervention of 80 or 120 mg/kg apatinib, both of which had statistically significant differences compared with the control group (*P* < 0.05). In the transplanted tumor tissue constructed with H524 cells, the expression levels of CD31, Ki67, and c‐caspase‐3 in the 80 mg/kg group were not significantly different from those in the control group (*P* > 0.05), while the expression levels of CD31 and Ki67 were significantly decreased, and the expression levels of c‐caspase‐3 were significantly increased in the 120 mg/kg group, which were statistically significant differences compared with the control group (*P* < 0.05) (Figure 5G‐L).

### 3.7 The clinical efficacy and safety

**FIGURE 6:**
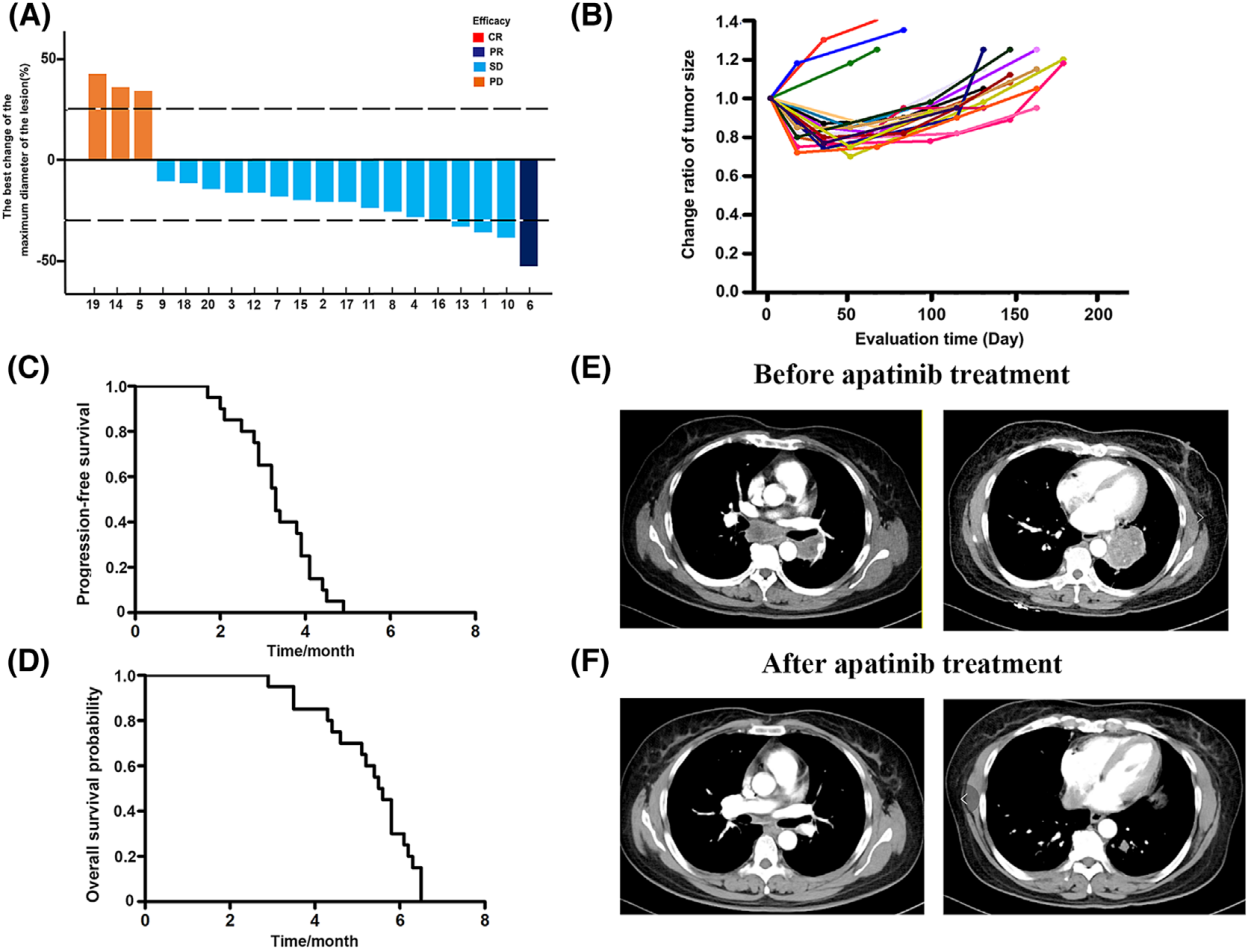
Clinical efficacy of apatinib for third‐ or above‐line treatment in patients with advanced small cell lung cancer. (A) Overall response of apatinib. (B) Changes of tumor size in individual patients after receiving the apatinib. (C) Kaplan–Meier survival curve of progression‐free survival. (D) Kaplan–Meier survival curve of overall survival. (E,F) The changes of chest CT before and after treatment with apatinib.

The basic information of the 20 patients with SCLC was shown in Table 1. All patients had measurable lesions. Following treatment, no patients achieved CR, one patient achieved PR, 16 patients achieved SD, and three patients developed lesions with PD. The best change of maximum diameter of lesion and the change ratio of tumor size were shown in Figure 6A,B. The patient survival curve is shown in Figure 6C,D, and the median PFS and OS are 3.3 and 5.6 months, respectively. Patients who achieved PR after 1 month of treatment with apatinib, a repeat spiral CT of the chest showed a significant reduction in the tumor lesion compared with the previous one, and the enlarged mediastinal lymph nodes were significantly reduced. The chest CT results of representative patients were shown in Figure 6E,F. In all patients, no grade 4 adverse reactions occurred, and only five cases of grade 3 adverse reactions occurred, accounting for about 25%. All grade 3 adverse reactions were tolerated, and treatment was not terminated. In the course of treatment, five cases of grade 3 adverse reactions were fatigue in two cases, hypertension in two cases, and proteinuria in one case.

## 4 DISCUSSION

Since the advent of antiangiogenic drugs, whether monotherapy or combined with other drugs, it can significantly increase the treatment effect and become a conventional treatment drug for advanced lung cancer.^15^, ^16^ The Beyond and E4599 studies confirmed the exact efficacy of bevacizumab combined with chemotherapy and greatly prolonged the survival of patients with advanced non‐SCLC.^17^, ^18^, ^19^ ALTER0303 study confirmed the effectiveness of anlotinib single‐agent third‐line therapy in the treatment of advanced lung cancer.^20^ However, the therapeutic effect of apatinib on lung cancer has been treated by few large clinical randomized controlled studies, and only some phase I and II clinical trials have also confirmed the partial efficacy. Most of the studies have mainly focused on advanced non‐SCLC.^21^, ^22^ There were few clinical and basic studies for SCLC. Tissue samples from clinical SCLC patients were selected to observe the relationship between VEGFR2 expression and patient prognosis in this study. The study confirmed that patients with high VEGFR2 expression had worse prognosis compared patients with lower VEGFR2 expression. So VEGFR2 may be a potential target for the treatment of SCLC.^23^ This study, from in vitro and vivo, confirmed that apatinib can inhibit the proliferation of SCLC cells expressing high VEGFR2, thus effectively controlling tumor progression.

Apatinib is a specific VEGFR2 inhibitor, and VEGFA‐VEGFR2 is an important signaling pathway for neoangiogenesis in almost tumors.^24^ Therefore, apatinib can reduce tumor neovascularization and control the growth of tumors, which is further confirmed by the better efficacy of apatinib in patients with gastric and colon cancer.^25^ However, it is not clear whether apatinib can directly act on the VEGRF2 of tumor cells and inhibit the cell proliferation by blocking the downstream signaling pathways.^26^ In vitro CCK‐8 test confirmed that apatinib effectively inhibited its proliferation in SCLC expressing high VEGFR2 and had a low IC_50_ value. At conventional oral doses of 500–750 mg/day, the blood concentration of apatinib in the human body can reach the preset concentration of in vitro tests, thus making it possible to treat apatinib in SCLC in the clinic.

The results of in vitro tests showed that a certain concentration of apatinib could inhibit the proliferation of small cell lungs expressing high VEGFR2 by inducing cell cycle arrest and promoting apoptosis and showing a concentration‐dependent manner.

**FIGURE 7:**
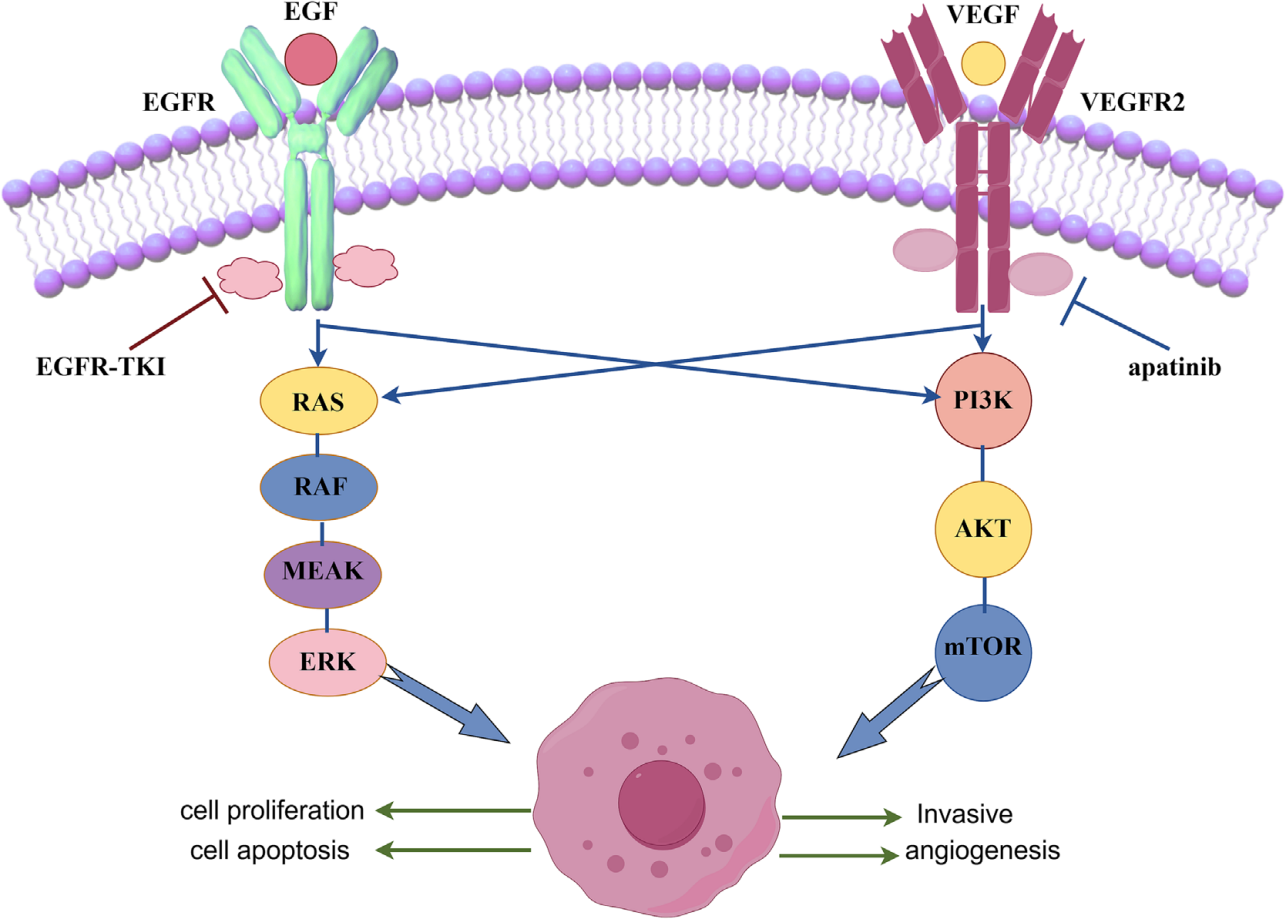
The intersection of the downstream signaling pathways of VEGF–VEGR2 and EGF–EGFR.

Although there is no neovascularization in vitro, apatinib acts only on the VEFGR2 of the cells, literature has reported that the intersection of VEGF–VEFR and EGF–EGFR signaling pathway exists downstream. Inhibition of VEGF–VEFR pathway can indirectly block the conduction of EGF–EGFR signaling pathway, thus achieving the effect of inhibiting cell proliferation.^27^, ^28^ The intersection of the downstream signaling pathways of VEGF–VEGR2 and EGF–EGFR were shown in Figure 7. If the cells were lowly expressed in VEGFR2, the effect of inhibiting cell proliferation in vitro is not apparent. As inferred from the results of in vitro cell tests, patients with SCLC with high expression of VEGFR2 may be the dominant population for apatinib treatment.

From the results of the animal test, the transplant tumor model constructed for H446 and H69 cells with relatively high expression of VEGFR2, either in the 80 or 120 mg/kg group, after a certain period of treatment, they all showed a certain treatment effect, and the effect was more obvious in the 120 mg/kg group, and the safety profile was also higher. No death or significant weight loss was found from the side effects of the drug. The transplant tumor model constructed for H524 cells with low expression of VEGFR2, although a significant tumor growth inhibition effect was only observed in the 120 mg/kg group; however, in the 80 mg/kg group, it also inhibited the tumor growth. This varies somewhat from the results of the in vitro cell assay, which, in in vitro cell assays, apatinib almost never inhibited proliferation in H524 cells. The difference during this period was that in the animal test, although the tumor cells themselves expressed low VEGFR2, there were blood vessels, which were mass expression of VEGFR2 in vascular endothelial cells. Therefore, a certain dose of apatinib can inhibit the production of tumor blood vessels, thus blocking the blood supply of the tumor and inhibiting the tumor growth. However, in in vitro cell test, due to the absence of tumor vessels, the proliferation inhibition of apatinib was not obvious for cells expressing low VEGFR2.

This study found that apatinib showed significant inhibitory effect on proliferation of SCLC with high expression of VEGFR2, both in in vitro cell test and animal test. In a small number of patients with SCLC, apatinib monotherapy also showed a certain therapeutic effect and was well tolerated. In these 20 patients with SCLC, ORR and DCR were 5% and 85%, and the PFS and OS are 3.3 and 5.6 months. Among the near‐term efficacy ORR and DCR were similar to the results reported in the literature, but the results of long‐term prognostic indicators PFS and OS were lower than the results reported in the literature, which was related to the inclusion of the patients in poorer physical condition and higher PS scores related.^29^ Studies have confirmed that apatinib can not only inhibit tumor growth by blocking neovascularization but also directly act on SCLC cells with high expression of VEGFR2, and inhibit tumor growth by promoting apoptosis and inducing cell cycle arrest. Future clinical studies can further expand the sample size and conduct randomized controlled studies to further confirm the efficacy and safety of apatinib in patients with SCLC. Due to the lack of effective targeted drugs for SCLC, immunotherapy is not effective, so antiangiogenic therapy combined with chemotherapy will become an effective treatment.^30^ How to further optimize the administration strategy of antiangiogenic and chemotherapy drugs may become an important means to improve the efficacy of SCLC patients.

## AUTHOR CONTRIBUTIONS

Mingtao Liu contributed the central idea, analyzed most of the data, and wrote the initial draft of the paper. Hui Li completed the animal test section. Ranran Guo completed part of the cell assay. The remaining authors contributed to refining the ideas, carrying out additional analyses, and finalizing this paper.

## CONFLICT OF INTEREST STATEMENT

None.

## ETHICS STATEMENT

All animal experiments were performed with the approval of Shandong University Animal Care and Use Committee. To minimize the pain and discomfort experienced by the animals during the experiment, using appropriate anesthetics, painkillers, and euthanasia. All patients signed informed consent prior to treatment. This study was conducted after approval of the hospitals Ethics Committee (ethical code: 2018166).

## Data Availability

The data that support the findings of this study are available from the corresponding author upon reasonable request.
